# Analysis of employment rate and social status in young adults with childhood-onset rheumatic disease in Catalonia

**DOI:** 10.1186/s12969-015-0026-8

**Published:** 2015-07-11

**Authors:** Ana Carolina Díaz-Mendoza, Consuelo Modesto Caballero, José Navarro-Cendejas

**Affiliations:** Vall d’Hebron University Hospital Pediatric Rheumatology Department, Barcelona, Spain; Center for research and teaching in economics (CIDE), Center for research and teaching in economics (CIDE)- National council of science and technology (CONACYT), Mexico City, Mexico

**Keywords:** Transition unit, Juvenile rheumatisms, Employment rate/social status

## Abstract

**Background:**

Rheumatic diseases of childhood, in particular juvenile idiopathic arthritis, are chronic conditions associated with considerable morbidity and mortality that can have repercussions on aspects of adult life. The aim of this study was to determine the employment rate and social status of patients with childhood-onset rheumatic disease attending a pediatric rheumatology transition unit.

**Methods:**

A census was taken of patients seen in the Pediatric Rheumatology Transition Unit of Hospital Vall d’Hebron (Barcelona, Spain). We collected demographic and clinical variables and determined the patients’ functional capacity. All patients seen during the period of September to December 2013 underwent a survey containing items related to their social situation, maximum academic level achieved, and working life. Correlations were sought between clinical variables associated with a poor prognosis and the patients’ job performance. The data were analyzed and compared with those of an age-matched cohort from the general population of Catalonia.

**Results:**

Of 130 patients included in the census, 96 responded to the survey. Steinbrocker grade III and IV disability (poorer functional capacity) (*p* = 0.0025) and longer disease duration (*p* = 0.017) were significantly related to greater difficulty getting a job. Patients with grade III and IV disability and those with more severe disease showed trends to having more problems carrying out work-related tasks. Our cohort included a higher percentage of students than the age-matched comparison population (50 % vs 24 %, respectively) (*p =* 0.0001); 82 % of patients had completed studies beyond the compulsory education level. The employment rate was lower in our patient cohort than in the comparison cohort (38.3 % vs 59.9 %) (*p =* 0.0001), whereas the percentage of unemployed was similar. Patients with milder disease had a higher probability of living with their parents up to a later age (OR = 3.2, 95 % CI 0.38-6.15; *p =* 0.029).

**Conclusions:**

Despite the advances in treatment, some patients with childhood-onset rheumatic disease encounter difficulties in their later social and working life. In our cohort, the time period needed to complete their studies tended to be longer, and incorporation into the workforce occurred at a later age. Our findings reinforce the idea that psychological support and vocational guidance are important factors in the management of these patients.

## Background

The prognosis of childhood-onset rheumatic diseases has changed considerably over time. In the past, many of these conditions were associated with high morbidity and mortality, whereas today most patients reach adulthood with little or no functional impairment. Nonetheless, there may be long-term physical, psychological, and social sequelae, including academic and work-related issues. Hence, it is important to know how the disease affects the life of these patients, to detect modifiable risk factors, and to identify patients who are more likely to have a poor outcome [1].

Various studies have examined the consequences of childhood-onset rheumatic diseases when patients reach adulthood, including the impact they may have on long-term health, psychosocial functioning, education, and employment. Studies published in the last two decades have reported divergent results, particularly in patients with juvenile idiopathic arthritis (JIA). These studies were performed in several countries having different cultures and healthcare systems, which may explain some of the differences.

The transition of pediatric patients into emerging adulthood can be defined as an active, multidimensional process [2]. The purpose of transition units is to provide guidance for patients and their families so that affected adolescents will become independent adults who actively participate in maintaining their health and are fully integrated in society [3]. Implementation of these units in pediatric rheumatology departments yields clear benefits for patients with childhood-onset rheumatic diseases, particularly JIA, as has been reported in several studies. For this reason, transition units should be recognized as a criterion of quality and be included in all rheumatology departments devoted to pediatric patients or adults [2].

The primary aim of this study was to determine the results regarding employment and social status in a cohort of patients attending the Pediatric Rheumatology Transition Unit of Vall d’Hebron Hospital (Barcelona, Spain). The secondary aims were to compare the employment rate in these patients with that of peers living in the same area having a similar cultural background and access to the same health system, and to investigate whether certain clinical variables may contribute to worsening the patients’ academic, employment, social, and family related-outcomes.

## Methods

### Population census

A census was taken of patients seen in our Transition Unit, a referral unit for patients with childhood-onset rheumatic diseases living in the geographic area of the Autonomous Community of Catalonia. The Transition Unit is staffed by the same professionals that manage these patients in the Pediatric Rheumatology Department.

All patients aged 15 to 35 years were included. Their clinical information was extracted from the hospital medical records database. The following demographic and clinical variables were recorded: age, sex, diagnosis, biological disease markers, years of follow-up, and current treatment. The International League of Associations for Rheumatology (ILAR) classification criteria were used in the diagnosis of patients with JIA.

### Employment survey

A questionnaire developed by the investigators requested data on the patients’ educational level (studies completed at the time of the survey), family situation, and employment status during the period of September to December 2013. Patients voluntarily answered the survey during a regular medical visit, and at the same visit, the attending physician determined their functional capacity according to the Steinbrocker classification scale: Class I, complete functional capacity with ability to carry out all usual work tasks without handicaps; Class II, functional capacity adequate to conduct normal activities despite the handicap of discomfort or limited mobility of one or more joints; Class III, functional capacity adequate to perform only a few or none of the tasks of usual occupation or of self-care; Class IV, Largely or wholly incapacitated, with the patient bedridden or confined to a wheelchair, permitting little or no self care [4]. Information was collected on demographics and the following items: the patients’ family situation, whether they lived alone or with their parents, the maximum academic level completed, current employment status, and their perception of how difficult it is for them to get a job or to carry out their work because of their disease. The questions related to the effect of the disease on employment had five possible answers ranging from “it does not affect me” to “it affects me very much”. Patients who did not attend a visit during the study period underwent the survey by telephone contact, and their functional status was determined from their medical records.

### Data from the general population of Catalonia

The employment data from our cohort were compared with those published in the *Enquesta a la Joventut de Catalunya 2012* (Catalonian Youth Survey, 2012), conducted by the Autonomous Government of Catalonia (GenCat). This is a demographic survey undertaken in 2012 in a Catalonian population aged 15 to 34 years. The sample included 3002 randomly selected individuals who underwent a personal interview in which data were collected on various items, including education and employment. Some items on the GenCat survey are reported by age groups: 16 to 19 years, 20 to 24 years, 25 to 29 years, and 30 to 35 years.

### Analysis of the social and family situation and impact of the disease in the employment setting

We investigated possible relationships of difficulties in getting a job and carrying out the work, the type of job, the patients’ educational level, and the social and family situation with the following variables: functional class, years of follow-up, type of disease, whether the patient was currently receiving biological treatment, and rheumatoid factor-positive or -negative disease.

The type of occupation was classified as unqualified (manual), qualified (not manual), or professional (requiring a university education). In a secondary analysis, patients were divided into severe or non-severe based on their disease. The following diagnoses were considered severe: systemic JIA, polyarticular JIA, and autoinflammatory syndromes. In the analysis of difficulties attaining and carrying out a job, patients were divided into two groups according to the length of follow-up (<15 years and >15 years). In the study of the impact of the disease on emancipation from parents, patients were divided into the same age groups as those used in the GenCat survey.

### Statistical analysis

A descriptive analysis was performed of the Transition Unit census and the data obtained in the patient survey. Exact tests for binomial variables were used to compare these data with those collected in the GenCat Catalonian Youth Survey, 2012.

Between-group differences were analyzed using a *t*-test for continuous variables (years of follow-up) and the Fisher exact test for categorical variables. For ordinal variables (functional class), a test for trends in proportions was calculated.

Multivariate logistic regression analysis was used to control for the potential confounding effect of age in estimating certain associations. Statistical significance was set at a p-value of <0.05. All statistical analyses were performed with Stata 13 (StataCorp, College Station, TX, USA).

## Results

### Census results

The census included 130 patients, 95 females (73 %) and 35 males (27 %), with a mean age of 22 years (range, 16–35). Mean (SD) follow-up was 14 (7.3) years. Among the total, 32 % (*n* = 41) of patients were currently receiving treatment with a biological disease-modifying antirheumatic drug (DMARD), 43 % (*n* = 56) were taking a non-biological DMARD, and 36 % (*n* = 47) were not under treatment. JIA was the most common condition in the cohort, accounting for 67 cases (51.5 %), followed by spondyloarthropathies with 28 cases (21.5 %), and autoinflammatory syndromes with 11 cases (8 %); 24 patients (18.5 %) had diseases other than those mentioned (*Others*). The spondyloarthropathy group included all patients with a diagnosis of spondyloarthropathy associated with inflammatory intestinal disease (inflammatory bowel disease-related arthritis), ankylosing spondylitis, reactive arthritis, and patients older than 16 years who had been diagnosed with enthesitis- related arthritis (ERA) in infancy, as 80 % of patients in this last group would be classified within the group of peripheral spondyloarthropathies using the new ASAS criteria [5, 6]. There were 42 patients (32 %) in the *severe* disease group and 88 (68 %) in the *non-severe* group (Table 2). The distribution according to Steinbrocker functional class was 97 patients class I, 19 class II, 10 class III, and 4 class IV.

Patients with JIA were distributed as follows: systemic JIA 13 % (*n* = 9), polyarticular JIA 33 % (*n* = 22), oligoarticular JIA 42 % (*n* = 26), psoriatic JIA 12 % (*n* = 8), and undifferentiated JIA 3 % (*n* = 2). Data on positive rheumatoid factor (RF) status and antinuclear antibodies (ANA) are presented in Table 1.

**Table 1:** Clinical and demographic characteristics of the Transition Unit cohort

Variables	*n* = 130
Demographics	
Women, n (%)	95 (73)
Men, n (%)	35 (27)
Age, y, mean (range)	22 (16–35)
Follow-up, y, mean (SD)	14 (7.3)
Clinical variables	
JIA, n (%)	67 (51.5)
ANA, n (%)	46 (35)
RF, n (%)	8 (6)
ERA, n (%)	28 (21.5)
Autoinflammatory sd, n (%)	11 (8)
Others, n (%)	24 (19)
Biological DMAR, n (%)^a^	41 (32)
Non-biological DMAR, n (%)	56 (43)
Untreated, n (%)	47 (36)
Steinbrocker class, n (%)	
I	97 (75)
II	19 (14.5)
III	10 (7.5)
IV	4 (3)

JIA, juvenile idiopathic arthritis; ERA, juvenile enthesitis related arthritis; ANA,antinuclear antibodies; RF, rheumatoid factor; sd, syndromes; DMAR, disease modifying antirheumatic drug ^a^Some patients receiving a biological DMAR were also receiving a non-biological DMAR

Among the autoinflammatory syndromes, there were 3 patients with childhood granulomatous arthritis (Blau syndrome), 2 mevalonate kinase deficiency (MKD)-related syndrome (also known as hyper-IgD syndrome), 5 patients with familial Mediterranean fever, and 1 with tumor necrosis factor receptor-associated periodic syndrome (TRAPS).

In the *Others* group, we highlight the following conditions: post-transplant osteoporosis (*n* = 4, occurring in patients whose main reasons for transplantation were primary biliary cirrhosis and cystic fibrosis, SAPHO (synovitis, acne, pustulosis, hyperostosis, and osteitis) syndrome (*n* = 1), connective tissue diseases *n* = 2 (scleroderma, SLE), vasculitis *n* = 2 (Takayasu, Behçet), and patients under study for suspected rheumatic disease (*n* = 9), that is, patients with a lupus-like condition or a suspected inflammatory autoimmune syndrome without characteristic mutations.

### Survey results

Of the 130 patients included, 96 responded to the survey. Sixty percent of patients who answered the survey had some type of JIA. The demographic characteristics, years of follow-up, disease severity, and functional status in this subgroup were similar to those of the total of patients surveyed. There were 19 patients in the 16–19 year-old age group, 45 in the 20–24 year-old group, 18 in the 25–29 year-old group and 14 in the 30–35 year-old group. With regard to the family and social situation, 39 % (*n* = 38) of the cohort were living independently from the family (alone or with other people, married or with a partner). The education level included 18 % with the compulsory level (studies completed at 16 years of age), 38 % with the post-compulsory level (completed at 18 years), and 43 % with a superior degree. As to the main occupation at the time of the survey, 38.3 % were working full time, 50 % were still completing their studies, and 11.7 % were unemployed.

Among the 36 patients who were working full time, the following job types were recorded: professional 50 % (*n* = 18), qualified 16.7 % (*n* = 6), and unqualified 33.3 % (*n* = 12) (Table 2).

**Table 2:** Family situation and occupational status of the Transition Unit cohort

Variables	*n* = 96
Family situation	
Live independently, n (%)	39 (41)
Live with parents, n (%)	57 (59)
Maximum academic level	
Compulsory level, n (%)	17 (18)
Post-compulsory level, n (%)	37 (38)
Superior level, n (%)	42 (44)
Occupation^a^	
Working, n (%)	36 (38)
Studying, n (%)	47 (50)
Unemployed, n (%)	11 (12)

^a^Some patients were in another occupational category

In response to the question of how difficult it is to get a job due to their disease, 70 % said that it did not affect them or affected them very little. Furthermore, 75 % answered that their disease did not affect or affected very little their ability to carry out their work.

### Findings from the Transition Unit of Hospital Vall d’Hebron cohort versus GenCat’s *Catalonian Youth* Survey cohort

The age distribution in the patient group was similar to that of the general population (GenCat cohort), but a comparison between the results found for the two cohorts showed some differences. The patient cohort contained a higher percentage of students than the general population in all age groups (50 % vs 24.4 %) (*p =* 0.0001). The percentage of persons working was lower in the patient cohort than in the general population (38.3 % vs 59.9 %) (*p =* 0.0001). No differences were found between the two groups in the percentage of unemployed persons (*p* = 0.6137). A detailed comparison of the data from the Transition Unit and GenCat surveys is presented in Table 3.

**Table 3:** Comparison of the Transition Unit cohort with the GenCat cohort from the general population by age group

Age group	Student, %			Employed, %			Unemployed, %		
	Patients	GenCat		Patients	GenCat		Patients	GenCat	
16-19	95.0	69.0			28.2		5.0	2.7	
20-24	56.5	25.3		30.4	64.1		13.0	10.6	
25-29	17.6	10.4		70.6	75.8		11.8	13.9	
30-35		8.0		84.6	79.2		15.4	12.9	
Total	50	24	*p* = 0.0001	38.3	59.9	*p =* 0.0001	11.7	9.8	*p =* 0.613

GenCat, Catalonian Youth Survey, 2012

### Analysis of difficulty in getting a job and carrying out the work

The statistical analysis showed no relationships between difficulties getting a job and a poorer functional class, (*p =* 0.0968), current biological treatment (*p =* 0.556), rheumatoid factor-positive disease (*p =* 0.236), disease type (*p* = 0.102), or disease severity (*p =* 0.114). There was, however, a trend to greater difficulty obtaining employment in patients with severe disease (40.91 % vs 17.39 % *p =* 0.070). Specifically, 75 % of patients with systemic JIA reported some difficulty or a great deal of difficulty obtaining work.

A statistically significant relationship was observed between longer duration of disease follow-up (>15 years) and difficulty in getting a job (*p* = 0.017); and a trend, albeit non-significant, to greater difficulty in carrying out the work (Table 4).

**Table 4:** Statistical relationships between the disease-related variables and occupation-related variables analyzed

	Type of disease	Severity	Functional class	Rheumatoid factor	Biological treatment	Duration of follow-up
Difficulty getting a job	*p* = 0.102	*p* = 0.114	*p* = 0.0968	*p* = 0.236	*p* = 0.556	*p* = 0.017
Difficulty carrying out the work	*p* = 0.232	*p* = 0.114	*p* = 0.0025	*p* = 0.175	*p* = 0.520	*p* = 0.542
Type of work	*p* = 0.534	*p* = 0.255	*p* = 0.811	*p* = 0.285	*p* = 0.560	*p* = 0.182
Highest academic level	*p* = 0.643	*p* = 1.000	*p* = 0.620	*p* = 0.301	*p* = 0.782	*p* = 0.96
Family situation	*p* = 0.140	*p* = 0.024	*p* = 0.580	*p* = 0.437	*p* = 0.177	*p* = 0.001

With regard to the impact of the factors studied on the ability to carry out the employment activities, patients in functional class III and IV had more problems in this regard than those with a better functional level (*p =* 0.0025). No relationships in this line were found for current use of biologics (*p* = 0.520), rheumatoid factor-positive disease (*p* = 0.175), or disease type. Nonetheless, in keeping with the findings for difficulties obtaining employment, there was also a trend to greater difficulty in carrying out work tasks in the group of patients with more severe disease (31.82 % vs 14.29 %; *p =* 0.114).

### Analysis of the type of employment

The type of work patients were doing (qualified, unqualified, or professional) showed no associations with their functional class (*p =* 0.81), biological treatment use (*p =* 0.560), or presence of rheumatoid factor-positive disease (*p =* 0.285). Examination of the type of work in relation to the severity of the disease showed that patients who had more severe disease had a non-statistical tendency (*p =* 0.255) to work at jobs that required less formal preparation (unqualified).

### Education level

There was no relationship between the patients’ academic level and any of the variables studied. Most patients (82 %) had completed studies beyond the compulsory education level.

### Family situation, social integration

Overall, the patients in our cohort lived for a longer time with their parents than the general population, particularly those with less severe disease. When the analysis was conducted by age subgroups, statistical significance persisted in the 20 to 24 year-old group (*p =* 0.016). In patients 30 to 35 years of age, 85 % lived independently. Patients whose diseases were considered *non-severe* had a higher probability of remaining with their parents until later adulthood (OD = 3.2, 95 % CI 0.38-6.15; *p =* 0.029]. Emancipation from parents was not affected by any of the other variables studied.

## Discussion

Childhood rheumatic diseases are complex, chronic disorders that raise concerns regarding the quality of life and social integration of affected patients when they reach adulthood. Despite considerable advances in the treatment of these conditions, these issues have remained a cause of discussion in the related literature over the last two decades.

The current study shows that 60 % of patients with childhood-onset rheumatic diseases attending a dedicated Transition Unit still required immunosuppressive treatment. This percentage is similar to the rate described by other authors [7], who found that many JIA patients have active disease as they pass into adulthood. Hence, they should continue to undergo regular medical monitoring, and this is the rationale for creating transition units as an integral part of pediatric rheumatology departments.

Few studies have focused on the relationships between education, employment, and disease in patients with childhood-onset rheumatic diseases, and many of them have shown contrasting data (Table 5). In 1997, Ruperto *et al*. reported quite optimistic data on the quality of life of JIA patients over a mean follow-up of 15 years. Nonetheless, the cohort was comprised of patients who had been diagnosed in tertiary hospitals within the first few years of disease onset and had received prompt treatment [7]. That same year, Peterson *et al*. reported greatly contrasting results from a cohort of patients in the United States. These authors described significantly poorer quality of life in JIA patients and a higher unemployment rate compared with controls, although no differences were found in the education level attained [8].

**Table 5:** Studies reporting social-functional results in patients with JIA compared to control patients over the last decades

Author	Year	No. patients	Years follow-up	Quality of life/functional ability instruments	Quality of life results	Education	Employment	Social status
Ruperto [7]	1997	290	14.9	SF-36	Good quality of life. No greater disability	No differences	No differences	No differences
				HAQ				
				CHAQ				
Peterson [8]	1997	44	24.7	HAQ	Lower than controls	No differences	Lower than controls	No differences
				SF-36				
Minden [9]	2002	215	16.5	HAQ	Good quality of life	Same or higher than controls	Same or higher than controls	No differences
				Steinbrocker				
Oen [18]	2002	392	13.5	CHAQ	Good quality of life	No differences	Lower than controls	ND
				Steinbrocker				
Packham [10]	2002	246	28.3	HAQ	Lower than controls	Higher than controls	Lower than controls	More single than married
Foster [11]	2003	82	21	HAQ	Poor quality of life	Higher than controls	Lower than controls	ND
				SF-36				
FlatØ [19]	2003	268	14.9	HAQ	Lower than the general population	No differences	Lower than controls	ND
				SF-36				
Arkela-Kautiainen [13]	2005	123	16.2	RAND-36	Same as controls	No differences	No differences	No differences
Gerhardt [14]	2008	45	12.64	SSPA	Same as controls	No differences	No differences	ND
Östlie [17]	2010	55	18.8	SF-36	Lower than the general population	Women, higher than controls Men, no differences	No differences	89 % do not live with parents
				HAQ				
				Ghq-20				

CHAQ, Childhood Health Assessment Questionnaire; GHQ-30, General Health Questionnaire; HAQ, Health Assessment Questionnaire; ND, no data; SSPA, Self-Perception Profile for Adolescents

At the beginning of 2000 in Germany, Minden *et al*. reported the findings of a cohort of JIA patients aged 20 to 35 years. The education level was similar or even higher than that of the controls and there were no differences in employment rates between the groups. Only 18 % of patients lived with their parents, reflecting their high level of self-reliance. Nonetheless, despite this evidence of favorable performance in both physical and social terms, many patients subjectively felt limited by their condition, especially those with active disease [9]. In a cohort of 246 JIA patients from the United Kingdom, Packham and Hall observed a considerable difference in the patients’ unemployment rate, being twice that of the general population, although the education level was higher in the patient group [10]. In 2003, these findings were corroborated in a study by Foster *et al*. [11]. Data from the most recent registry of childhood-onset rheumatic diseases in Germany indicated that employment rates in men and women with JIA were lower than the control population and also lower than patients with adult-onset rheumatoid arthritis or spondyloarthropathies [12]. In contrast to these findings, a study performed in 2005 in Finland reported similar results for education, employment, and social functioning between JIA patients and controls [13]. More recently in the United States, Gerhardt *et al.* described a JIA cohort between 18 and 20 years of age that showed no differences in education level or employment status compared with age-matched peers [14].

The reason for the high unemployment rates despite good academic performance is uncertain, but it has been suggested that in addition to the degree of functional involvement, psychosocial factors may also be important for integrating these patients into the labor market [14]. In the study by Malvija *et al.* in 2012, the education level achieved at ages 16 and 18 years predicted the type of job a JIA patient would have in future life, and the degree of impairment was directly related to the extent of future unemployment the patient might face. This study underscores the importance of career guidance and vocational planning for these young people through their educators, with extension of this dialogue to health professionals, as JIA is a complex disease with a highly variable functional outcome [15].

In our cohort, the employment rate was lower than that of the general population of Catalonia, especially in the group aged 20 to 24 years, using the results from the Catalonian Youth Survey as reference. In addition, our results suggest that incorporation of these patients into the labor market occurs at a later age. One possible explanation for this finding may be that young people with these conditions need more time to complete their studies, as has been suggested by other authors in studies that show greater absenteeism from school due to the disease [9]. It is also possible that our patients strongly believe they must reach a higher academic level to compete on the work market and therefore, they continue their studies for a longer time.

As to the impact of the disease on employment status, we observed that the percentage of unemployed in our survey was similar to that recorded in the 2012 Catalonian Youth Survey in all age groups and at different education levels (9.8 % in the general population vs 11.7 % in the cohort). The results of the Gencat survey, a socio-demographic study used here as the reference for the comparisons, may differ somewhat from those of surveys specialized in employment, such as the Encuesta de Población Activa (Economically Active Population Survey); therefore, it may only partially reflect the true impact of the economic crisis on the general population. The GenCat survey was chosen for the comparisons because it provides specific data about the population of Catalonia, whereas other surveys include the overall Spanish population.

The results from our cohort show a higher percentage of students in almost all the age groups than in the general population. The academic level of most patients was post-compulsory or superior. Assuming the limitations in representativeness of our survey due to the sample size, our findings are consistent with those of several studies that found no differences in the academic performance of these patients [8, 9, 11, 13, 14, 16] and even those that observed a higher educational level compared with controls [10, 17].

As would be expected, patients in functional class III or IV and those with longer disease duration reported significantly greater difficulty carrying out tasks related to their jobs, despite the limited samples. No correlation was found between the other variables studied (eg, type of disease, use of biological treatment, or rheumatoid factor-positive disease) and the different measures of social and employment-related integration. The trend showing that patients with severe disease graded by diagnosis have greater difficulty getting a job and doing the work leads one to presume that with a larger sample, this factor would have reached statistical significance. Among the diseases studied, systemic JIA was associated with poorer outcomes.

With respect to the social and family situation, patients considered to have non-severe disease lived with their parents up to a later age. The cohort was divided into severe and non-severe according to the diagnosis and attending to the general prognosis of these diseases, not according to the functional capacity. However, the fact that a disease is considered non-severe does not imply that the patients’ functional capacity is good, which may in part explain this finding. Furthermore, as occurs in other populations of children with chronic disease, the parents may tend to overprotect them, which could result in the patients’ reaching independent adult maturity at a later age.

To our knowledge, this is the first study focusing on the later employment status of patients with childhood-onset rheumatic disease in Catalonia. It includes various diseases and a wide age-range in adulthood, examining correlations regarding incorporation into the workforce with the use of a survey. The number of years of follow-up of our patients is comparable to that of other cohorts from studies in Europe and the United States that have examined similar issues. Furthermore, the patients all attended the same unit, which uses uniform diagnostic criteria and treatment protocols that have included biological therapy since the year 2000.

The main limitation of the study is the relatively small size of the cohort, which precluded specific study of the JIA subgroup in relation to employment and social status. Pediatric rheumatic diseases are not highly prevalent and multicenter studies would be needed to achieve patient samples comparable to those of studies in adults. The study group is heterogeneous, but this is the type of real-life patients we see in our transition unit. In addition, few patients in our cohort were in functional class III-IV, which may have biased the results to a somewhat better outcome. Lastly, because the study was conducted within our daily clinical practice with a limited time per patient, we did not have the opportunity to use a patient-reported quality of life instrument, which would have provided information on the patients’ view of how the disease affects them.

In summary, despite the advances in the management of patients with childhood-onset rheumatic diseases that have occurred over the last decade, a considerable number require periodic follow-up as they reach adulthood. The cohort surveyed in our Transitional Unit had good functional status overall, but those with moderate to severe functional impairment had more difficulty getting a job and carrying out the tasks required. Furthermore, the employment rate of our patients was lower than that of the comparison cohort from the general population although the majority had attained a good academic level.

### Conclusion

In our setting, patients diagnosed with childhood onset rheumatic diseases showed a trend to live independently and join the labor market at a later age than their peers in the general population, whereas their academic level was similar or higher. Only those with a longer disease duration and greater functional impairment reported difficulty in getting a job or carrying out the tasks required. The findings from this study lend support to the widely accepted strategy of multidisciplinary management of these patients, in which psychological support and vocational guidance should have a prominent role.
